# Treatment of the Acute Bronchial and Pneumonic Diseases of Children

**Published:** 1896-01

**Authors:** James Orr

**Affiliations:** Terrell, Texas


For Texas Medical Journal.

### TREATMENT OF THE ACUTE BRONCHIAL AND PNEUMONIC DISEASES OF CHILDREN.

BY JAMES ORR, M. D., TERRELL, TEXAS.

Read before Terrell Medical Society.

THE SOCIETY having chosen as the subject for discussion to-day the lung diseases of children in winter, and assigned me the task of opening the same, I have selected the above heading for the purpose of calling your attention to two diseases embraced thereunder, with particular reference to treatment in their acute stages.

I trust the members of the society will pardon my digression sufficiently to call their attention to the importance of a correct diagnosis in these, as in all other complaints they are called upon to treat. The doctor who pronounces every case of somnolency congestion of the brain, every case of sore throat diphtheria, or every cough and sore lungs pneumonia, is either inexcusably ignorant or culpably dishonest; in either case doing no credit to the profession, while the medical attendant who mistakes the collapse of broncho-pneumonia for the crisis of lobar pneumonia commits an irretrievable error. Therefore I would call your particular attention to the importance of differential diagnosis, fully endorsing the writer who said, "He who diagnoses correctly, finds little trouble in treating."

The first subject to which I shall call attention is the common coryza, or catching a bad cold, that we see so frequently in young children, in which the whole trouble is of an acute character and located in the nasal air passages and larynx. In the treatment of such cases I usually administer a mild purgative that will act quickly, using mercury if I think it indicated. I order the patient given a warm bath and kept in a warm moist room where it can breahte steam if possible. As medicine I give codeia and atropia in homeopathic doses, say for a child of six months 1-40 gr. codeia and 1-500 of atropia every hour till the little patient falls into a quiet sleep. These medicines are usually combined with spirits nitre dulce and syr. yerba santa co., or pruni virginiana, and I have rarely found but few doses necessary. For local treatment I direct camphorated vaseline or suet applied freely over the nose and frontal region, and sometimes spray, a 5% solution of antipyrine, gently into the anterior nares.

I can not call to mind a single instance in which this treatment has failed to relieve the patient in a few hours.

In bronchitis we have a graver complication to meet, yet if seen in the acute stage I think it will be found to yield quite promptly. Here again I endeavor to place the patient in a warm, well ventilated room, the air of which is rendered moist by escaping steam; everything calculated to worry or excite the little sufferer is, as far as possible, removed, and for treatment opiates are my main stay, which I give in infinitesimal doses, often repeated. In cases where there is rapid, hard pulse, like a cord under the finger, hot, dry skin and bright eyes, I find aconite acts beneficially, and in this class of cases a good combination is tinct. ipecac et opii composita, tinct. aconita and spirits nitre dulce in syr. prun. virgin. When obtainable, I prefer using Flemming's tinct. aconita, as being uniformly reliable, and I would lay particular stress on the superior action of medicines when given to children in very minute doses, frequently repeated, till the desired result is obtained.

In very young children I have found Aubergier's syrup lactucarium almost a specific in the acute and subacute form of bronchitis. With this preparation, combined with infinitesimal doses of liquid Dovers powder, I have seen the happiest results, the fever, quick pulse, cough and restlessness rapidly giving way to true convalescence. It may not be amiss to call attention to the fact that in the primary or acute stages of bronchitis expectorants should never be given.

When the acute symptoms of bronchitis have passed, and the secondary or subacute stage has set in, good hygiene and properly selected expectorants will effect a speedy cure. One word as to expectorants and cough syrups, viz: That it is very important to remember the therapeutic action of the various remedies usually employed, if we would obtain the most satisfactory results; for instance, that ammonia increases and limes decreases expectoration; that senega is a powerfully sedative expectorant; that yerba santa and pruni virgin. are not expectorants, but *vice versa*, and so on. Keeping these points in view, I usually treat cases where there is a hard cough, giving some pain but with little expectoration or signs of secretion, with a prescription containing codeia, muriat ammonia, syr. senega, syr. tolut. suitable to the age of the child. In older children I find a drop or two of chloroform added to the night doses very beneficial.

In cases where the cough is less painful and secretion free I use codeia lactophosphate of lime, fl. ext. pruni virginiana and syrup yerba santa co. If the secretion is very free I sometimes add eucalyptus or syr. pix liquida to this prescription. In cases where the discharge assumes the character of a bronchorrhœa, I use syr. iodide calcis or vin iodotan, of Nourray, with equal and perfectly satisfactory results. In very young children, unable to cough and clear the bronchial tubes, I labor to limit the expectoration as rapidly as possible.

In the treatment of the graver forms of bronchitis, whether we call it catarrhal fever, influenza or la grippe, the symptoms being practically the same, I make no deviation from the plans laid down above. I regard opiates as the sheet anchor and the other remedies as adjuvants.

In all cases of systematic depression, I promptly resort to the use of alcoholic stimulants, usually preferring it given in the form of milk punch.

Croupous or lobar pneumonia is not a frequent disease among children in this country, but cases are sometimes met, and should be easily recognized. In the management of these cases I try to have the little patient placed in a warm, well ventilated room where after a warm bath, I have his entire chest enveloped into several thicknesses of cotton batting sewed between soft flannel, which is to remain unchanged, if possible, till the crisis of the disease. Having gotten him settled in the most quiet and comfortable place obtainable, I move his bowels well with a mercurial, followed by caster oil, if necessary, and order bicarb. potass., spts. of nitre dulcis, and syrup of prun. virginiana every two hours during the day, but under no circumstances is he to be awakened to receive medicine. In case of pain, restlessness, or distressing cough I use an opiate, preferring codeia, and when this drug fails to relieve febrile symptoms and control the pulse promptly, I carefully administer aconite. Remembering that the tendency to death in pneumonia is from asthenia, I watch the heart carefully, that I may commence the use of alcoholic stimulation as soon as indicated. This disease usually terminates by crisis in from six to nine days, at which time prompt stimulation is necessary; after which I order some very mild tonic in conjunction with out door exercises, expecting perfect resolution and speedy recovery. It sometimes happens that the disease does not subside by crisis, and that in its stead we have tardy resolution, emaciation, peevishness and debility, in which case if the child is large enough to swallow capsules I always give iodoform in full sized doses in conjunction with maltine and wine of cocoa. In case capsules can not be swallowed I give vinum iodotan or syrup of iodid iron if the latter is indicated. In one very rebellious case I got good and prompt results from the use of Donovan's solution in connection with the tonic treatment named.

The next disease to which I will call your attention is broncho-pneumonia, which we find described under various headings, principally, however, as capillary bronchitis, lobular and interstitial pneumonia. This grave disorder is fortunately rare in this country. That it is usually a secondary affection occurring in weak or debilitated children, and has a tendency a chronicity, indicates the line of treatment as stimulant and tonic. It is a source of congratulation to myself that so few cases have fallen under my care, for one finds little satisfaction in treating patients requiring so much care and yielding such bad results.

The first step is to place the patient in the most favorable hygienic surroundings possible, and to supply it with palatable nourishment in concentrated form. I then direct the little sufferer's body shampooed twice a day with diluted alcohol and have it taken into the sunshine in the open air when the weather and its condition will permit. I promptly order alcoholic, stimulants in the shape of egg-nog, milk punch, wine whey or toddy, according to the age of the patient, or as it can be best given and borne. As a cough mixture I order carb. ammonia, tinct. sanguinaria fl. ex. yerba santa co., and syrup of lactophosphate lime, which I give every two or three hours, and add small doses of codeia to the night dose if indicated. Some alterative is essential, and I have found nothing superior, if indeed equal, to vinum iodotan of Nurray, heretofore mentioned, which I give in maximum doses three times daily. Under this plan of treatment the last four cases of broncho-pneumonia treated by me have recovered in periods from thirty to sixty days, and in neither case did

I have abscess, hepatisation or atelactasis as a resulting complication or injury.

Before concluding I wish to call attention to two forms of distressing cough in children. One is the night cough which usually comes only after several hours sleep, is at first a little hack, then a hard, dry distressing cough, occurring in paroxysms at frequent intervals, for varying periods, then to disappear until about the same time the next night. This cough is purely reflex, and can be promptly cured in nine cases out of ten by some of the simpler remedies for post-nasal catarrh.

The other is that deep, hard dry cough, distinctly bronchial, and heard at all periods of the day. It may or may not follow an acute attack of bronchial or pulmonary trouble, and is always worse in winter and after exposure to inclement weather. Such cases I always treat with fl. ex. lippia Mexicana and syr. lactophosphate of lime in full doses, finding it almost a specific.

